# Two Novel Variants of *WDR26* in Chinese Patients with Intellectual Disability

**DOI:** 10.3390/genes13050813

**Published:** 2022-05-02

**Authors:** Jiacheng Hu, Mingming Xu, Xiaobo Zhu, Yu Zhang

**Affiliations:** 1 Department of Neurology, Xinhua Hospital Affiliated to Shanghai Jiao Tong University School of Medicine, Shanghai 200092, China; hujiacheng@sjtu.edu.cn (J.H.); qwww_991076@163.com (M.X.); 18757434714@163.com (X.Z.); 2 Shanghai Jiao Tong University School of Medicine, Shanghai 200025, China

**Keywords:** Skraban-Deardorff syndrome, intellectual disability, *WDR26*, Chinese

## Abstract

Skraban-Deardorff syndrome is a rare autosomal dominant genetic disease caused by variants in the *WDR26* gene. Here, we report two Chinese patients diagnosed with Skraban-Deardorff syndrome caused by novel de novo, heterozygous pathogenic *WDR26* variants c.977delA (p. 12 N326Ifs*2) and c.1020-2A>G (p. R340Sfs*29). Their clinical features were characterized by intellectual disability (ID), developmental delay, abnormal facial features and the absence of early-onset seizure, which expands the phenotype spectrum associated with Skraban-Deardorff syndrome. By comparing our cases with current reported cases of *WDR26*-related intellectual disability, we suggest that developmental delay, particularly in speech, and facial features including rounded palpebral fissures, depressed nasal root, full nasal tip and abnormal gums, represent the prominent clinical phenotypes for diagnosis of Skraban-Deardorff syndrome. Together, *WDR26* variants and 1q41q42 deletions should feature prominently on the differential diagnosis of ID with distinctive facial features.

## 1. Introduction

Skraban-Deardorff syndrome, a subtype of *WDR26*-related intellectual disability (ID), is a newly recognized form of ID which is caused by variants in the *WDR26* gene [1]. Containing 14 exons, the *WDR26* gene is located in the chromosomal region 1q42.12. The WDR26 protein includes WD-40 repeats, conserves LisH and CTLH domains, and comprises 661 amino acids. In human fetal tissues, the expression of WDR26 is highest in brain and skeletal muscles, and lowest in liver, lungs and heart [1,2,3,4]. As of now, a total of 26 variants in the *WDR26* gene have been reported, in which three cases were identified through whole exome sequencing (WES) without detailed clinical description. The 26 variants reported include eight missense variants, eight frameshift variants, seven stop-gained variants and three splicing variants (Table S1) [1,5,6,7,8,9]. Clinical manifestations of Skraban-Deardorff syndrome are heterogeneous and cover a broad spectrum of symptoms that can range from neurologic manifestations to non-neurologic ones. These include ID with delayed speech, seizures, gait abnormalities, developmental delay, characteristic facial features and multiple structural anomalies [1]. Detailed descriptions of the clinical phenotypes are limited due to the limited number of reported cases, as only 23 Skraban-Deardorff syndrome patients from Europe and the United States have been reported [1,5,6]. Here, we report two cases of Skraban-Deardorff syndrome with novel heterozygous variants in the *WDR26* gene and are, to the best of our knowledge, the first to describe the clinical features of Chinese patients.

## 2. Materials and Methods

### 2.1. Ethics Statement

This study was performed with the approval of the Ethics Committee of Xin Hua Hospital (XHEC-D-2022-058). Written informed consent were obtained from patients’ parents.

### 2.2. Genetic Studies and Variant Assessment

Genomic DNA was extracted from peripheral blood of the patients and their family members with the QIAAMP DNA blood mini-kit (QIAGEN, Valencia, CA, USA). The capture kit was XGen Exome Research Panel v1.0 (Integrated DNA Technologies, Coralville, IO, USA). The process of WES was the same as previously reported [10]. Output fastq files were then aligned to the hg19 human reference genome using BWA-0.7.10. Variant calling was conducted according to the GATK best practice workflow, and snpEff was used to annotate the VCF files. We excluded the variants which were observed to have a frequency >1% in the 1000 Genomes Project, gnomAD and the Exome Variant Server (EVS) database and those at a frequency over 5% in the local database (containing 6000 exomes) from the list of candidate variants. Filtered variants were further analyzed using autosomal recessive, autosomal dominant, and X-linked inheritance patterns. The variants were classified according to the guidelines of American College of Medical Genetics and Genomics (ACMG). CNV calling was performed with a CS-CNV pipeline. An XHMM and CNV kit were applied to call CNVs of the sample [11]. Sanger sequencing was used to validate the *WDR26* variants discovered by WES. Family members including parents and siblings were tested for the identified variants to support pathogenicity and segregation with the disease. PCR amplification sequences were produced by Sangon Biotech Co., Ltd. (Shanghai, China) to analyze the variant. Detection of genomic copy number variations (CNVs) was performed using the Affymetrix CytoScan HD array (Affymetrix, Santa Clara, CA, USA). The spliceAI website (https://spliceailookup.broadinstitute.org/, 7 March 2022) was used for prediction of the splicing identified.

## 3. Results

### 3.1. Clinical Features of Case 1

**Figure 1:**
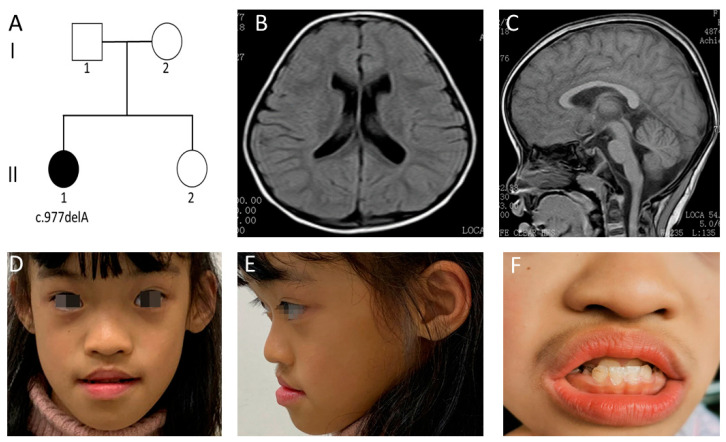
Clinical manifestations and auxiliary examinations of the case 1. (**A**) Family pedigree of case 1. (**B**,**C**) Brain MRI scans demonstrating mild enlarged ventricles and an absence of other structural brain anomalies (T1-weighted image). (**D**–**F**) Distinctive facial features were identified. These included arched eyebrows, rounded palpebral fissures, a depressed nasal root, full nasal tip and anteverted nares. She had a wide mouth, decreased Cupid’s bow, short philtrum, full lips, an anterior reverse bite, enamel hypoplasia, widely spaced teeth, and abnormal gums.

An 11-year-old Chinese girl was the first of two children born to nonconsanguineous Chinese parents (Figure 1A). After a full-term pregnancy, the patient was born at 40weeks of gestation through caesarean section. The birth weight and length were 2900 g (<50 centile) and 50 cm (>50 centile), respectively. While no feeding difficulties were noted, the patient suffered from severe diarrhea at the ages of 7, 12 and 18 months. However, no hypotonia or abnormalities of the respiratory, ENT (ear, nose, and throat), ophthalmological, musculoskeletal, or cardiac systems were detected. 

Mild developmental delay was first noted at the age of 12 months when the patient first began to utter simple words like “papa”. She could not sit until age of seven months and was unable to walk without support until 20 months. Neurodevelopmental status was determined using Gesell Developmental Schedules at 29 months. These evaluations showed that the developmental quotient (DQ) of four domains fell within the range for mild to moderate retardation. These included scores of 51, 58, 49, 37 and 57 for adaption, gross motor, fine motor, language, and personal/social functions. She scored a severely low DQ in the language function domain (37, DQ < 40). Despite several years of frequent, specialized training which started from the age of 29 months, her mental development levels had not improved significantly. No history of regression was observed. At the age of 39 months, magnetic resonance imaging (MRI) of the brain demonstrated mild enlarged ventricles, without other structural brain anomalies (Figure 1B,C). 

Upon the first physical examination in our hospital at the age of 11 years, she had a height of 143 cm (<50 centile), weight of 27.6 kg (<10 centile), and head circumference of 49 cm (<3 centile). Her cognitive development was severely delayed as she could only speak in short sentences (five to six words) and had difficulty in understanding speech. While her motor development was mildly delayed, she could walk and run independently. However, she still needed support for daily activities such as toilet habits and dressing. Her social living ability was measured using the Infant-Junior Middle School Student’s Ability of Social Life Scale and it generated a standardized score of 5. This suggests extremely deficient social living abilities, which included deficiencies linked to self-help, locomotion, occupation, communication, socialization, and self-direction. Distinctive facial features were identified, including arched eyebrows, rounded palpebral fissures, a flattened nasal bridge, a broad nasal tip and anteverted nares. She had a wide mouth, decreased Cupid’s bow, short philtrum, full lips, an anterior reverse bite, enamel hypoplasia, widely spaced teeth, and abnormal gums (Figure 1D–F). She was also noted to have an abnormal spastic gait and mild hypotonia. Neither febrile nor non-febrile seizures were observed in this patient, and we did not find abnormal electroencephalograms at any of her follow-ups. Her personality was described as friendly, without autism. However, she showed repetitive hand movements such as the biting of fingernails and the unconscious touching of ears. It is worth noting that, from the age of six years, the patient has had suggestive symptoms of masturbation that occur five to seven times a week. These symptoms include tightly crossing her legs and clenching the leg muscles in order to create pressure on the genitals. This clinical symptom has not been reported before. Upon examination, her thyroid function, metabolic screening and chromosomal karyotype analyses were all normal. She is educated at a local special education school.

### 3.2. Clinical Features of Case 2

**Figure 2:**
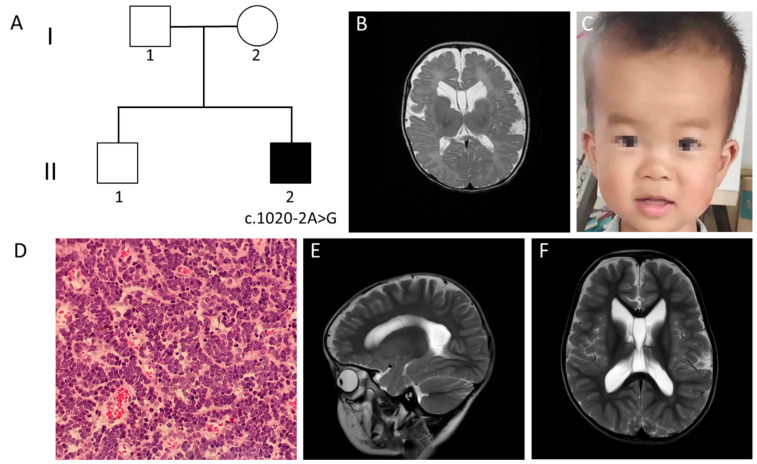
Clinical manifestations and auxiliary examinations of the case 2. (**A**) Family pedigree of case 2. (**B**) Brain MRI scans at 42 days demonstrated widened brain ventricle, deep brain sulci and subependymal cyst (axial plane, T2-weighted image). (**C**) Distinctive facial features were identified. These included protruding forehead, rounded palpebral fissures, large irises, depressed nasal root, full nasal tip and abnormal gums. (**D**) Hematoxylin and eosin staining of surgical resection demonstrating highly cellular tumor with round nuclei and little cytoplasmic differentiation (original magnification ×200). (**E**,**F**) Follow-up brain MRI scans at three years of age still demonstrated enlarged brain ventricle, deep brain sulci and subpendymal cyst (sagittal and axial plane, T2-weighted image).

A three-year-old Chinese boy was born at 37 weeks through caesarean section as the second child from non-consanguineous Chinese parents (Figure 2A). His birth weight and length were 3900 g (>85 centile) and 50 cm (50 centile), respectively. 

The patient first came to our hospital at the age of five months due to abnormal findings in an MRI which was conducted in local hospital. The brain MRI showed a widened brain ventricle, deep brain sulci and a subependymal cyst (Figure 2B). Physical examination showed that he had a length of 65 cm (<50 centile), weight of 8.5 kg (>85 centile), and head circumference of 44 cm (>85 centile). His electroencephalogram showed abnormally slow waves. Other previous tests included normal karyotype, tandem mass spectrometry and ultrasonic cardiogram. In addition, no abnormalities of respiratory, ENT, ophthalmological, musculoskeletal or cardiac systems were found. However, hypotonia was detected. 

The patient could roll over at 6 months, sit at 7 months, and walk and utter words at 12 months. At the age of three, he could only pronounce sentences with five to six words. Gesell Developmental Schedules were conducted and showed that the DQs of the patient in the domains of adaption, gross motor, fine motor, language and social behavior were 62, 75, 75, 58 and 83, respectively. Mild developmental delay was reported, especially in language. His personality was described to be friendly, without autism. Seizures had not appeared until recently. Abnormal facial features were found, including a protruding forehead, rounded palpebral fissures, large irises, depressed nasal root, full nasal tip and abnormal gums (Figure 2C). 

His medical history was remarkable for posterior fossa medulloblastoma. Physical examination at the age of five months showed an enlargement of head circumference. After identification of genetic abnormality associated with the *WDR26* gene, a follow-up MRI was conducted at the age of 11 months, which revealed space-occupying lesions in the vermis of the cerebellum. The tumor was totally removed by surgery. Pathological findings showed classic medulloblastoma (Figure 2D). The tumor was classified into the sub-type of sonic hedgehog (SHH), which was decided by nanoString-based subgrouping [12]. The patient was treated with ten courses of chemotherapy without radiotherapy. Follow-up examination at the age of three showed that the tumor had not recurred, while a brain MRI still demonstrated widened brain ventricles, deep brain sulci and a subependymal cyst (Figure 2E,F). 

### 3.3. Genetic Analysis

**Figure 3:**
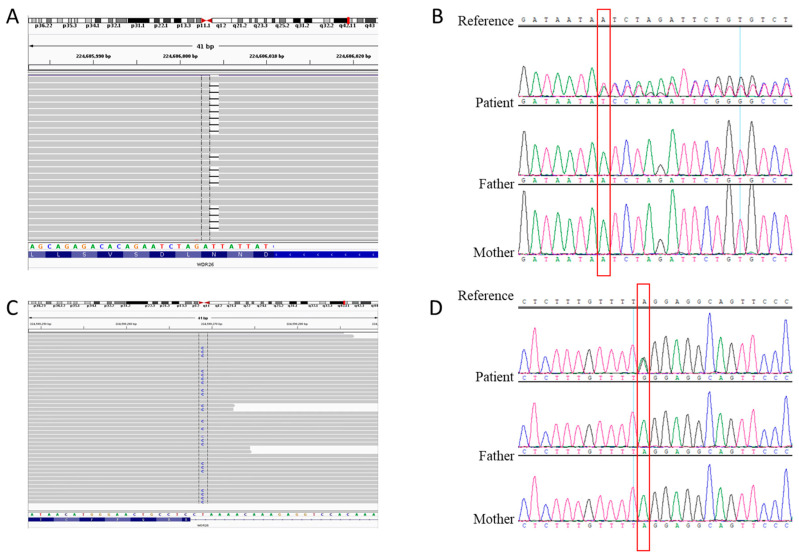
Detection and analysis of the *WDR26* variants in our patients. (**A**) The IGV illustration of the c.977delA variant in the *WDR26* gene. (**B**) Sanger sequencing results for the c.977delA variant in case 1. The parents were variant negative. (**C**) The IGV illustration of the c.1020-2A>G variant in the *WDR26* gene. (**D**) Sanger sequencing results for the c.1020-2A>G variant in case 2. The parents were variant negative.

Exome sequencing was performed to detect possible variants which cause the disease. A novel heterozygous c.977delA (p. N326Ifs*2) variant was identified in the *WDR26* gene (NM_025160.6) of case 1 (Figure 3A,B). This variant results in a frameshift in the sixth exon of the *WDR26* gene. According to the ACMG guidelines for variant interpretation [13], it is evaluated as pathogenic (PVS1 + PM2 + PM6). A novel heterozygous c.1020-2A>G (p. R340Sfs*29) variant was identified in the *WDR26* gene (NM_025160.6) of case 2 (Figure 3C,D). This variant was located at the canonical sequences of AG-acceptor nucleotide. This variant is assessed to be pathogenic (PVS1 + PM2 + PM6). 

Results of the WES were confirmed by Sanger sequencing, and the variants were not detected in the healthy parents and sibling in each family (Figure 3B,D). Frequency analysis showed that these two variants did not present in the 1000 Genomes Project, EVS or the gnomAD databases. Although they could have been inherited through parental germline mosaicism, the likelihood of this reoccurring was estimated to be extremely low. Notably, no variant was identified in the *FBXO28* gene at a minor allele frequency of ≤5%. Similarly, other previously proposed candidate genes for chromosome 1q41-q42 microdeletions were also not found. CNV analysis did not detect deletions in the 1q41-q42 region.

## 4. Discussion

We reported two pediatric cases of Skraban-Deardorff syndrome caused by novel de novo, heterozygous variants in the *WDR26* gene, c.977delA and c.1020-2A>C, respectively. Skraban-Deardorff syndrome is a rare disease characterized by developmental delay, abnormal gait and recognizable facial phenotypes. It is estimated that, in European and American patients, the frequency of *WDR26* variants is about 1 in 1500 for individuals presenting with ID and about 1 in 2000 for individuals manifesting all indications [1]. However, the prevalence in Asian patients remains unspecified, as variants in Asians have been rarely reported [14]. Importantly, from over 2500 ID cases investigated in our laboratory, two patients with the *WDR26* variants have been identified. In this regard, the prevalence of Skraban-Deardorff syndrome in Asian patients would be similar to that in European and American patients. 

There were several differences in the clinical phenotypes between our patients and the reported cases with *WDR26* gene variants. According to previous studies, and except for an 18-month-old infant in whom the seizures might not yet have started, seizures were found in all the other patients with Skraban-Deardorff syndrome caused by the *WDR26* haploinsufficiency. Therefore, early-onset seizure was considered as a stereotypical symptom in Skraban-Deardorff syndrome, with the age of onset ranging from neonatal to seven years old [1,5,6]. Since seizures were never detected in both of the cases described in our patients who were three years old and 12 years old, respectively, it could be concluded that the occurrence of seizures does not necessarily accompany with dysfunction of WDR26. Several facial features that are common in patients with *WDR26* variants, including full cheeks as a child and a protruding upper lip, were also not observed in our patients. At present, little correlation has been found between the *WDR26* gene variants and their associated disease phenotypes. 

Importantly, similarities in phenotypes between our cases and previous cohorts have been identified as well. In our cases, among several evaluated domains of neurological development, the domain of language is prone to be most severely impaired, while other domains including motor and social behavior would be relatively normal. By reviewing the cases reported, we propose that, instead of seizure, severe language disorder should be considered as the stereotypical symptom of Skraban-Deardorff syndrome. Adequate and timely language therapy would contribute to better prognosis in children with Skraban-Deardorff syndrome.

The *WDR26* gene is one of the major genes located in the 1q41q42 region, which is prone to be disrupted in the chromosome 1q41-q42 deletion syndrome (OMIM612530) [15]. There are several overlapping clinical features in individuals with Skraban-Deardorff syndrome and those with chromosome 1q41-q42 deletion syndrome. Therefore, WES and chromosomal microarray analysis should include screening for *WDR26* variants and 1q41-q42 microdeletions in ID patients with distinctive facial features. Genotype-phenotype correlation studies have been performed using data from previously reported cases associated with 1q41-q42 microdeletions and *WDR26* variants [1,2,5,6,15,16,17,18,19,20,21,22,23,24,25,26,27,28] (Table 1). Twenty-three cases associated with *WDR26* variants were identified in European and American patients noted to have ID, seizures and distinctive facial features [1,5,6]. Twenty-nine cases of Asian, European and American patients diagnosed with 1q41-q42 microdeletions were identified. Their clinically distinguishing features were also found to include developmental delay, ID, and seizures [1,2,15,16,17,18,19,20,21,22,23,24,25,26,27,28]. Comparing the clinical phenotypes of the Skraban-Deardorff syndrome cases to those with chromosomal 1q41-q42 microdeletions, we could conclude that delayed speech, ID, gait abnormalities and characteristic facial features including rounded palpebral fissures, depressed nasal root, full nasal tip and abnormal gums are core symptoms of Skraban-Deardorff syndrome. In addition, because of a single gene variant rather than the deletion of multiple genes, several patients with Skraban-Deardorff syndrome showed relatively mild clinical manifestation as case 2 showed in this study. We compared the clinical phenotypes of cases with Skraban-Deardorff syndrome to those with chromosome 1q41-q42 deletion syndrome cases (Table 1). The result shows that an abnormal gait is a trait in 82.35% (14/17) of Skraban-Deardorff syndrome patients, but is present in less than 20% (3/16) of the patients with chromosome 1q41-q42 deletion syndrome. In contrast, hypotonia, nail hypoplasia, abnormalities of the digits, gastrointestinal dysfunction and skeletal abnormalities, which occur frequently in chromosome 1q41-q42 deletion syndrome patients, are less common in Skraban-Deardorff syndrome patients. These differences demonstrate that chromosome 1q41-q42 deletion syndrome cases have more severe and diverse clinical manifestations, which include the presence of cataracts, short stature, microcephaly, and multiple structural anomalies [1,2,5,6,15,16,17,18,19,20,21,22,23,24,25,26,27,28].

There are several limitations in our study. Firstly, a long-term follow-up study is necessary to assess the progression of disease in the two cases. Secondly, genotype-phenotype correlations associated with Skraban-Deardorff syndrome were not found due to the insufficient number of samples. Thirdly, a direct assessment of the functional consequences of the variants was not conducted in this study, and would therefore be a point of interest for future investigation.

The c.977delA (p. N326Ifs*2) variant detected in the *WDR26* gene of case 1 is a frameshift variant in exon 6 that gives rise to a premature termination codon. In case 2, the c.1020-2A>G (p. R340Sfs*29) variant identified is considered pathogenic because the A to G transition affects the −2 residue at the 3′ acceptor splice site. This variant may lead to the loss of the original acceptor and the gain of a new acceptor at the ninth nucleotide upstream according to spliceAI. In both patients, the predicted protein would be truncated and loss-of-function; alternatively, the level of the mRNA produced would be relatively low. WDR26 is widely expressed in human tissues, with the highest expression levels found during developmental stages in brain and skeletal muscles [1,2,3,4]. This may indicate the underlying mechanism in which ID and abnormal gait are found in most cases. In vivo and in vitro experiments are needed to explore the pathogenic mechanism of *WDR26* haploinsufficiency in the nervous system in the future. 

In conclusion, we described two Chinese cases of Skraban-Deardorff syndrome with novel *WDR26* variants in detail. Clinical features of the patients included intellectual disabilities with delayed speech, developmental delays, gait abnormalities and characteristic facial features, including rounded palpebral fissures, depressed nasal root, full nasal tip and abnormal gums. However, early-onset seizure was not detected in both of our cases, which have been considered as a representative phenotype of Skraban-Deardorff syndrome. By comparing our cases with the reported cases of *WDR26*-related ID, we provided an expansion on the phenotype spectrum of this rare disease. *WDR26* Haploinsufficiency and 1q41q42 deletions should be screened for differential diagnosis of ID with dysmorphic features.

## Figures and Tables

**Table 1:** Clinical features of patients with *WDR26* variants or chromosome 1q41-q42 deletion syndrome.

Features	Patient 1	Patient 2	*WDR26* Variants [1,5,6] (*n* = 23)	Chromosome 1q41–q42 Deletion Syndrome [1,2,11,12,13,14,15,16,17,18,19,20,21,22,23,24] (*n* = 29)
Developmental delay or intellectual disability (ID)	+	+	23/23	27/27
Limited speech	+	+	23/23	8/8
Seizures	–	–	22/23	17/27
CNS structural anomalies	+	+	15/22	18/24
**Hypotonia**	+	+	14/20	9/21
**Abnormal gait**	+	+	14/17	3/16
Happy and/or friendly personality	+	+	18/19	4/4
Autistic and/or repetitive behaviors or posturing	+	–	11/17	3/3
Facial features				
Coarse facial features	+	+	15/23	18/22
Full cheeks as a child Large irises or rounded/short/slanting palpebral fissures	– + (rounded)	+ + (rounded)	18/21 15/23 (rounded)	9/10 7/12 (slanting)
Abnormal eyebrows	–	–	11/23	6/13
Depressed nasal root	+	+	12/23	18/22
Anteverted nares	+	–	15/23	12/16
Full nasal tip	+	+	19/23	14/20
Prominent maxilla	–	–	16/23	10/17
Protruding or full, tented upper lip	–	–	16/23	13/18
Wide mouth	+	–	12/17	8/17
Decreased cupid’s bow	+	–	11/23	12/15
Widely spaced teeth	+	–	18/21	9/10
Abnormal gums	+	+	13/19	8/9
Hypertelorism	–	+	5/15	13/21
Ophthalmologic abnormalities	–	–	10/17	3/8
**Nail hypoplasia**	–	–	3/13	6/6
Short stature	–	–	3/21	5/19
**Digit abnormalities**	–	–	5/14	8/10
**GI difficulties**	+	–	11/16	7/8
**Orthopaedic disorders**	–	–	13/17	15/15

NA: not available; Features noted in bold represent the discrepant features.

## Data Availability

The data that support the findings of this study are available from the corresponding author.

## Supplementary Materials

The following are available online at https://www.mdpi.com/article/10.3390/genes13050813/s1, Table S1: Full list of 28 variants identified in *WDR26* gene.
